# HoloPointer: a virtual augmented reality pointer for laparoscopic surgery training

**DOI:** 10.1007/s11548-020-02272-2

**Published:** 2020-10-23

**Authors:** Florian Heinrich, Florentine Huettl, Gerd Schmidt, Markus Paschold, Werner Kneist, Tobias Huber, Christian Hansen

**Affiliations:** 1 Faculty of Computer Science, University of Magdeburg, Magdeburg, Germany; 2 Research Campus STIMULATE, Magdeburg, Germany; 3 Department of General, Visceral and Transplant Surgery, University Medicine of the Johannes Gutenberg University Mainz, Mainz, Germany; 4 Department of Surgery, Hospital St. Marienwörth, Bad Kreuznach, Germany; 5 Department of General and Visceral Surgery, St. George Clinic Eisenach, Eisenach, Germany

**Keywords:** Virtual pointer, Medical augmented reality, Laparoscopic surgery training, Head pointing

## Abstract

**Purpose:**

In laparoscopic surgery training, experts guide novice physicians to desired instrument positions or indicate relevant areas of interest. These instructions are usually given via verbal communication or using physical pointing devices. To facilitate a sterile work flow and to improve training, new guiding methods are needed. This work proposes to use optical see-through augmented reality to visualize an interactive virtual pointer on the laparoscopic.

**Methods:**

After an interdisciplinary development, the pointer’s applicability and feasibility for training was evaluated and it was compared to a standard condition based on verbal and gestural communication only. In this study, ten surgical trainees were guided by an experienced trainer during cholecystectomies on a laparoscopic training simulator. All trainees completed a virtual cholecystectomy with and without the interactive virtual pointer in alternating order. Measures included procedure time, economy of movement and error rates.

**Results:**

Results of standardized variables revealed significantly improved economy of movement (*p* = 0.047) and error rates (*p* = 0.047), as well as an overall improved user performance (Total *z*-score; *p* = 0.031) in conditions using the proposed method.

**Conclusion:**

The proposed HoloPointer is a feasible and applicable tool for laparoscopic surgery training. It improved objective performance metrics without prolongation of the task completion time in this pre-clinical setup.

**Electronic supplementary material:**

The online version of this article (10.1007/s11548-020-02272-2) contains supplementary material, which is available to authorized users.

## Introduction

Advantages of minimally invasive surgery are a reduced risk of infection, a shorter hospital stay and recovery time. However, these benefits are accompanied by technical difficulties like having spatially separated monitors instead of direct vision on the patient and the operation site [1]. This mentally demanding psychomotor task complicates hand-eye-coordination and leads to perceptual issues regarding the correct position of instruments and patient anatomy [2–4].

Therefore, training and learning are crucial in order to compensate for these issues. Besides technical skills, guiding novice surgeons toward regions of interest on the laparoscopic camera screen, such as the Ductus cysticus or the Arteria cystica, and teaching them subsequent viewing and working directions has become a major part of this training process in minimally invasive surgery [5]. The importance of obtaining this professional vision has been identified by various research groups [6, 7]. Training in this domain mostly involves the following of trainers’ verbal commands, gestures or pointing directions [8]. However, these guiding methods are often impractical, inapplicable or inefficient. Voice commands may be ambiguous, while hand gestures may not be noticed and be hard to interpret, as well. Moreover, pointing with physical devices requires the trainer to have an available free hand during assistance. Additionally, this may compromise operation workflow and sterility in case of a non-simulated training.

To solve these issues, we propose to use augmented reality (AR) to superimpose the vision of trainees with useful annotations guiding them to relevant areas of interest. Likewise, the same technique can be used by trainees to facilitate communication with their supervisor in case of further inquiry. To this end, we developed a virtual AR pointer application using the mixed reality glasses Microsoft HoloLens, which allowed for a desired hands-free interaction using head pointing and voice recognition. This *HoloPointer* was evaluated by 10 surgeons during a virtual cholecystectomy on a commercially available surgical training simulator.

## Related work

Previous work in this domain presented different surgical pointing tools. Table 1 provides an overview of these systems. A first published example showed Ursic et al. [9] describing the idea of placing a common laser pointer inside a sterile latex housing. The hand-held pointer was then used to indicate landmarks on the laparoscopic video screen to facilitate communication.

**Table 1:** Key findings and issues of pointing systems developed for laparoscopic surgery

References	Year	Interaction	Tracking	Findings	Issues
Ursic et al. [9]	1997	Hand-held laser pointer	–	Reduced risk of self-contamination, frustration and inaccuracy compared to conventional pointing	No hands-free use possible
Jayaraman et al. [10]	2009	Head pointing	Optical Tracking Camera	Improved instruction efficiency (less task completion time) compared to conventional pointing	Use of tracking camera limits working area
Prescher et al. [11]	2014	Laparoscopic camera	–	Improved instruction efficiency (less task completion time) compared to conventional pointing	Laparoscopic camera position cannot be changed without moving the pointer
Chetwood et al. [12]	2012	Gaze position	Eye Tracking Camera	Improved instruction efficiency (less task completion time, reduced error rates) compared to conventional pointing	Users cannot change their gaze focus without moving the pointer
Ward et al. [13]	2012	Head pointing	Optical Tracking Camera, Inertial Tracking	Improved interaction performance (less total pointer movement, smoother trajectory) compared to commercial pointer system	Use of tracking camera limits working area
Trejos et al. [14]	2015	Head pointing	Optical Tracking Camera, Inertial Tracking	Less hand pointing required, improved instruction efficiency (subjective questionnaires) compared to conventional pointing	Use of tracking camera limits working area
Feng et al. [15, 16]	2018	Hand gestures	Depth Camera	Improved economy of instrument movement, improved gaze behavior (more concentrated and more clustered fixations)	No hands-free use possible

In contrast, Jayaraman et al. [10] proposed a hands-free solution for such a pointer, arguing that usually both hands of surgical trainers are already occupied. An optical tracking camera was used to detect the position of a fiducial marker attached to a surgical mask of the trainer. Head movement was then transferred to movement of a pointer on the video screen. Results of a user study, during which a trainer guided 20 trainees toward points of interest on a laparoscopic box trainer, suggest an improved efficiency compared to a verbal guidance only condition. The method required the trainer to stay at a fixed position in front of the used tracking camera, that was attached to the trainer’s monitor.

A similar method was used by Prescher et al. [11] to evaluate a pointer which was integrated into the laparoscopic camera. An attached fluorescent dot was moved together with the camera and could thus be used to direct trainees to specific target positions. Within a localization task, this pointer could be shown to improve guiding efficiency. However, the fixed connection of camera and pointer limited the system’s usability.

Another pointing tool, described by Chetwood et al. [12], tracked a trainer’s gaze position and superimposed it onto the laparoscopic video screen. This method was also shown to reduce completion times and errors for laparoscopic box trainer tasks compared to a verbal guidance condition. Yet, this approach required trainers to constantly focus their gaze on intended target structures, thus constraining its use.

Ward et al. [13] developed another wireless hands-free surgical pointer for minimally invasive surgery. Sensor fusion between inertial measurement units attached to a head-mounted device and an optical tracking camera on the laparoscopic monitor was used to transfer head movements to the position of a pointer on the video screen. A comparison with a commercially available hands-free pointing system showed advantages of their method in terms of less total pointer movement and smoother movement curves. Later, Trejos et al. [14] described their experiences of using this method in the operating room for surgical instruction during laparoscopic cholecystectomies. Based on subjective questionnaire data of involved medical staff, using the pointer facilitated the instruction communication compared to conventional methods.

Finally, Feng et al. [15, 16] presented a virtual pointer that was controlled by hand gestures and superimposed the laparoscopic video screen. This pointer was developed with the purpose of facilitating the adoption of professional vision. Results of two user studies show more concentrated and more clustered fixations of trainees compared to a standard condition and thus suggest the pointers capabilities of modifying the user’s gaze. Moreover, some objective improvements, e.g., economy of movement, were shown in later runs of the study. However, this approach required at least one unoccupied hand of the trainer in order to manipulate the pointer’s position.

## HoloPointer

Most of the previous attempts to develop surgical pointer applications are based on directly superimposing the laparoscopic video signal with a virtual pointer object and displaying both on the monitor. This, as well as the individually chosen interaction modalities, limits to some extent the potential and capabilities of these solutions. Physical pointer devices [9] occupy at least one hand of their user. Attaching a pointer to the laparoscopic camera [11] complicates camera navigation and restricts the field of view. Other approaches use external tracking cameras attached to the laparoscopic monitor [10, 12, 15] and thus constrain the working area to these cameras’ fields of view. Moreover, past approaches only supported the visualization of one pointer controlled by the trainer and have not regarded the potential of providing trainees with similar means of communication.

We attempt to solve these issues using optical see-through AR HMDs. Such self-contained systems do not require external tracking hardware, provide the user with different interaction techniques and are able to communicate wirelessly. Our prototype was developed using the mixed reality glasses Microsoft HoloLens (first generation, Microsoft Corporation, USA), because they were positively evaluated for clinical use and showed promising results in the past [17–19].

Built-in cameras enable the recognition of hand gestures and are used in conjunction with inertial measurement units to accurately locate the HMD in space [20]. This allows for head movements to be tracked correctly, thus also enabling head pointing. Additionally, the HoloLens has microphones used for speech recognition. We decided to focus on head pointing to manipulate the position of a pointer object. This allows for a hands-free interaction. In contrast to gaze position tracking, it limits the user’s focus of vision only to a certain extent and the user may briefly lose the optical focus from the laparoscopic monitor (during change of instruments) without immediately changing the pointer’s position.

**Fig. 1:**
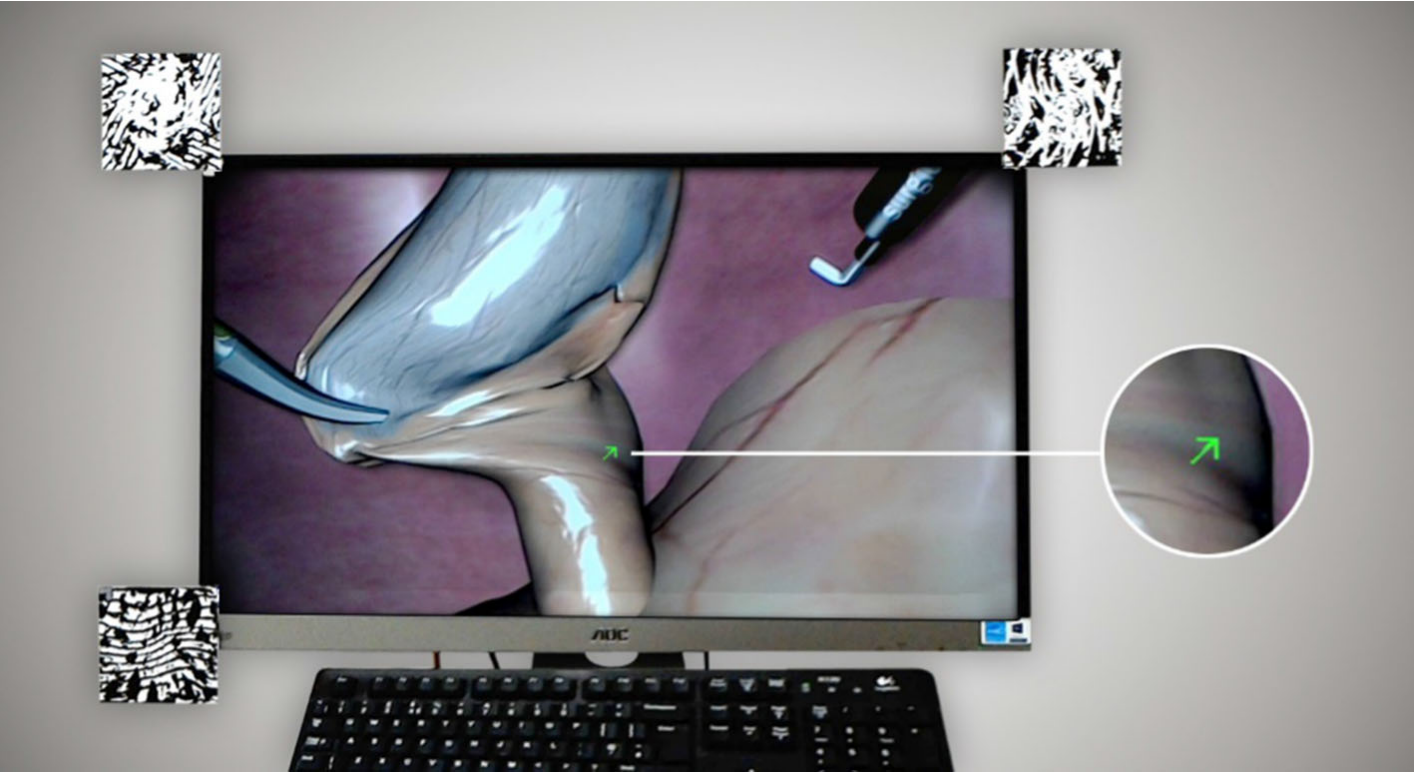
HoloPointer as seen through the HoloLens. A green arrow indicates a point of interest during simulated laparoscopy surgery training. Image markers on the monitor were used for screen registration

The virtual pointer application was developed using the game engine Unity (Unity Technologies, USA). In this application, the laparoscopic video screen is defined by three image markers attached to the corners of the screen (see Fig. 1). These markers are initially detected using the Vuforia AR SDK (PTC Inc., USA). Afterward a plane approximating the video screen is calculated from the measured corner positions. This registration process needs to be conducted only once at program start. Afterward, the local position of the virtual monitor plane remains stable due to the HoloLens’ spatial tracking functions [21].

**Fig. 2:**
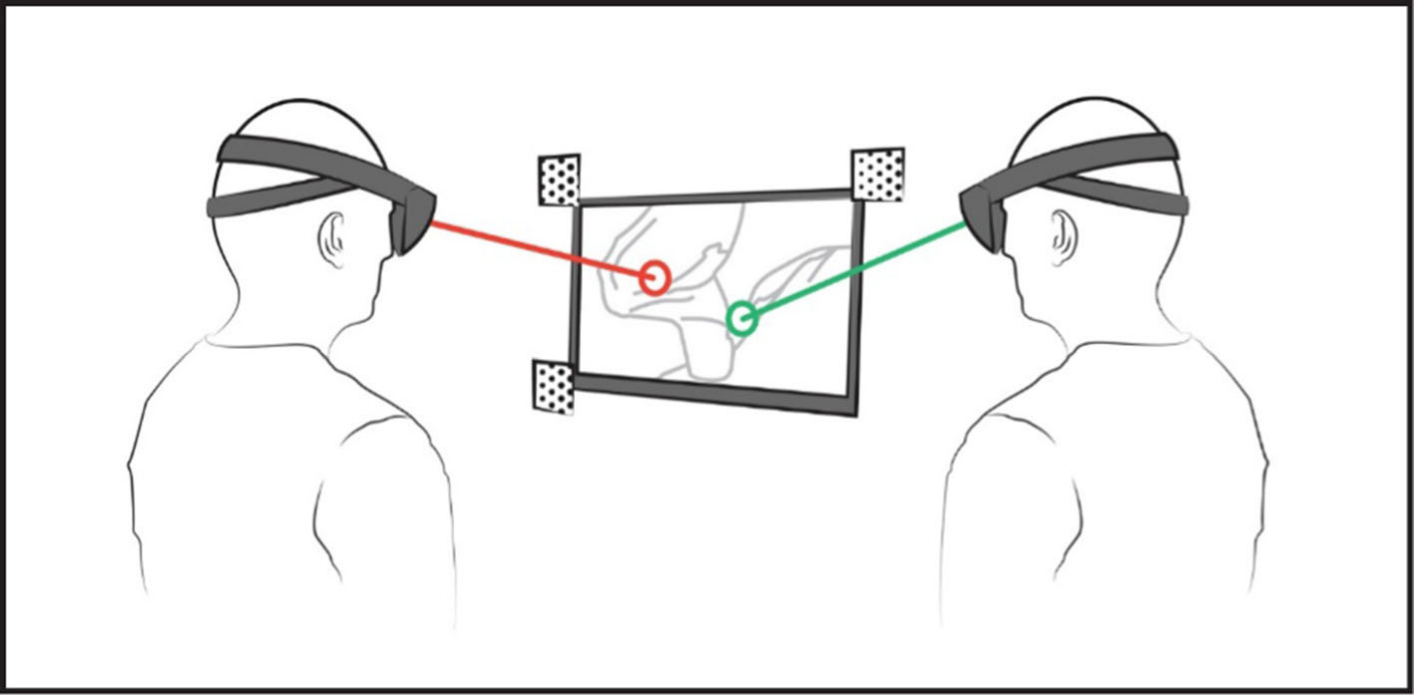
Illustration of the HoloPointer. Virtual pointers are rendered at the intersection of two users head orientation and a registered monitor. HMDs communicate wirelessly to visualize both pointers for both users

During each update frame, a ray is cast from the wearer’s head position along the head’s frontal principal direction. The intersection of this ray and the virtual video screen plane is then used to calculate the position of an image-based virtual pointer object. Non-linear interpolation is used to smooth the transition between the last pointer position and current intersection point. Smaller movements are more affected by this than larger ones, to compensate for slight trembling of the head. This new pointer position is then transformed to a local two-dimensional coordinate system defined by the image markers attached to the video screen. This 2D position is finally transmitted wirelessly to any additional HoloLens worn by a different user. At the same time, data is received from these other pointer applications. Their sent 2D coordinates are then transformed to local 3D space using the detected image marker positions and applied to respective additional pointer objects. That way, every user is able to see her/his own pointer together with any connected users’ pointers within the same coordinate frame. This is illustrated by Fig. 2. Moreover, image markers used for screen registration can be attached to multiple monitors. Therefore, it is also possible that users of different locations can communicate with each other via the pointer.

The pointer application was extended by the possibilities for placing stamps, i.e., static copies of the user’s own pointer at its current position, and drawing lines. These objects can also be transmitted to other running applications. Both features are activated via key phrases using built-in speech recognition. Likewise, the color, size, shape and orientation of the user’s pointer can be varied. Based on clinical feedback, the starting size was set to 1.25 cm × 1.25 cm for the arrow and ring shapes and to 0.7 cm × 0.7 cm for the dot shape. Additional voice commands include the capabilities to show and hide selected pointers and to remove or hide lines or stamps. Figure 1 shows the resulting pointer application, called HoloPointer hereinafter, in use.

## Applicability and feasibility for laparoscopic training

In the pre-clinical user study, the HoloPointer was assessed for its use on a virtual reality laparoscopic simulator. The study was carried out as a cross-over, within-subject design study with a standard condition consisting of verbal and gestural communication and a condition with additional HoloPointer support.

### Participants

One senior surgeon was invited to conduct the training during the study as trainer. A total of ten members of the surgical department, seven junior residents (4 female; training year 1–6), two senior residents and one consultant (3 male) were asked to participate in the role of trainees. Cholecystectomies are considered difficult for a student without any exposure to laparoscopy. Thus, residents and fellows have been selected for the study. Six residents reported to have performed below 20 laparoscopic cholecystectomies, while the other surgeons reported to have performed over 50. Only one participant was left handed.

### Apparatus

**Fig. 3:**
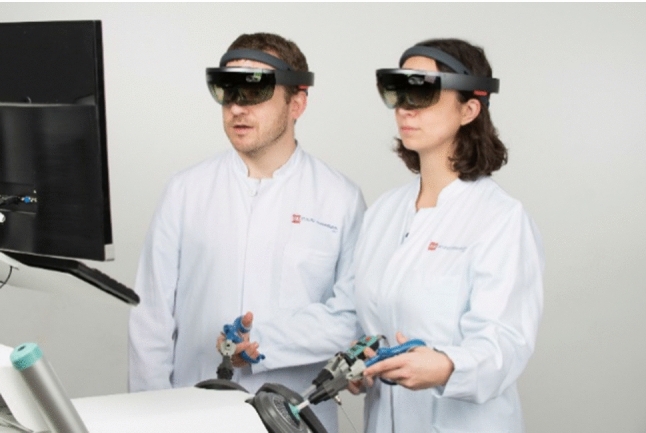
Experimental design. The surgeon (left) guides a resident during virtual laparoscopic surgery using an optical see-through HMD

The experimental setup consisted of two Microsoft HoloLens HMDs with running HoloPointer applications. Image markers were attached to the virtual reality laparoscopic training simulator (LapSim, software version 2015, Surgical Science, Sweden). The simulator was operated with a camera and two grasper joysticks representing a laparoscopic camera, a grasper, and a coagulation and dissection electrode inside the virtual simulation, as well as a foot pedal. Figure 3 shows two physicians using the HoloPointer during training on the used simulator.

### Tasks

The trainees (*n* = 10) were asked to conduct the vessel preparation step of the simulator’s cholecystectomy procedure module twice. This included the identification and preparation of the cystic artery and cystic duct, followed by sufficient clipping and dissection of these structures. The same surgeon assisted all virtual cholecystectomies. The trainer was instructed to guide the trainees either with verbal commands or manual corrections (standard condition) or to additionally use the HoloPointer to indicate regions of interest and to convey gaze guidance associated with professional vision.

### Measures and variables

To assess the HoloPointer’s usefulness and feasibility, objective measures derived from the training simulator were of interest as dependent variables. Assessed items were time (s), instrument path length (m), and angle (°) for left and right, blood loss (ml), number of missing or fatal clips (n), coagulation damage (%), dissected volume (ml), number of vessels ripped or burned (n), number of missing cuts (n), and number and time of instruments out of sight (*n* and *s*, respectively). All available simulator data dimensions were standardized with the formula *z* = *x* − *μ*/*σ*, where *x* is the raw score, *μ* is the mean and σ is the standard deviation of the parameter. Resulting *z*-scores were then combined to summarizing variables. For the *procedure time* variable, the *z*-score of the total time was used, representing means to measure efficiency. *Errors* were calculated by the sum of *z*-scores of blood loss, number of missing or fatal clips, coagulation damage, dissected volume, number of vessels ripped or burned, number of missing cuts, and number and time of instruments out of sight. The sum of instrument path length, and angle for left and right was used to calculate the variable *economy of movement.* Additionally, an overall *summary z*-score was calculated.

### Procedure

First, demographic information was acquired for each participant, e.g., gender, level of qualification. Then, they were instructed about the experimental procedure and their tasks. Afterward, the subjects were asked to put on the HoloLens and needed knowledge regarding the device and the HoloPointer was explained. Each participant completed two subsequent vessel preparations, one with a visible HoloPointer of the trainer and one without it. The sequence of conditions was alternated between participants. The HMDs were worn in both runs to ensure similar conditions. After completion of the second trial, participants were asked to answer five custom questions regarding subjective feedback on the HoloPointer on 5-point Likert scales. The questions are listed in Table 3. After the study we collected objective performance data from the simulator. Moreover, the trainer’s pointer position and the trainees’ gaze positions were recorded for each time frame during the study.

## Results

After conducting the study, we decided to exclude the measured data from trials with the two senior residents and the consultant. These participants had more than 6 years of work experience and had higher hierarchical positions than the trainer within the surgical department. These status-related differences together with the high level of prior knowledge resulted in less effects of trainer guidance in both experimental conditions.

**Fig. 4:**
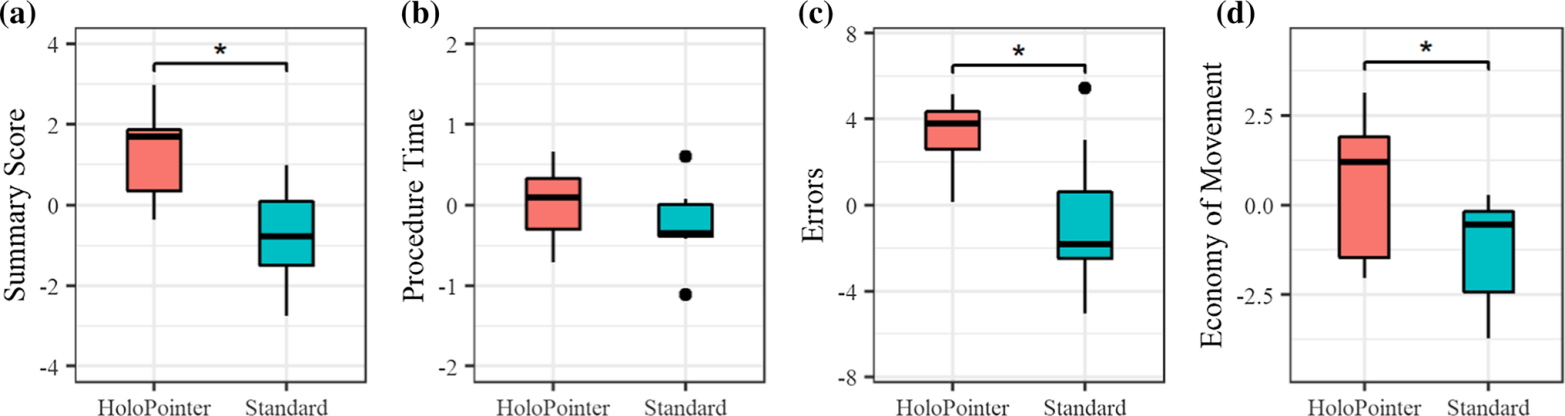
Effects of the guiding modality factor on the *z*-scores of standardized variables **a** summary score, **b** procedure time, **c** errors and **d** economy of movement. Asterisks represent statistical significance

Variables derived from standardized *z*-scores were statistically analyzed using Wilcoxon signed rank tests, as parametric test requirements could not be assumed to be fulfilled. Table 2 summarizes the tests’ results and Fig. 4 visualizes respective effects. Statistically significant effects were shown regarding the overall *summary score* variable, indicating that use of the HoloPointer was generally advantageous compared to the standard condition. The guiding modality also had statistically significant effects on the *errors* variable, indicating that using the HoloPointer resulted in a reduction in errors recorded by the simulator. Moreover, the HoloPointer provided for a statistically significantly improved *economy of movement*. Consulting raw simulator output data reveals that this were probably due to a significantly reduced path length of the right hand’s instrument (*p* = 0.012, calculated by pairwise *t* test). No statistical significance could be shown for the *procedure time*.

**Table 2:** Summary of Wilcoxon signed rank test results (*α* < 0.05)

Variable	*V*	*p*	Significance	*r*	Effect size
Summary score	27	0.031	*	0.81	Large
Procedure time	21	0.297		0.39	Medium
Errors	26	0.047	*	0.75	Large
Economy of movement	26	0.047	*	0.75	Large

* represent statistical significance

Table 3 summarizes subjective assessment results regarding the proposed HoloPointer method. In general, participants reacted positively to the application. It was reported to be helpful and participants said to have followed its instructions. Wearing the HoloLens resulted in some degree of discomfort. The darkening effect posed by the tinted lenses of the device did not seem to disturb the participants and their field of view was only marginally obstructed.

**Table 3:** Subjective feedback questionnaire. Scores represent verbal anchors: 1-strongly agree, 2-agree, 3-neutral, 4-disagree, 5-strongly disagree. Mean scores and standard deviation are reported

ID	Question	Mean score
Q1	The AR glasses were comfortable to wear.	3.5 ± 0.9
Q2	My field of view was not obstructed by the HMD.	2.5 ± 0.5
Q3	The darkening effect caused by the tinted lenses was not disturbing.	2.2 ± 1.1
Q4	The support of the HoloPointer was very helpful for the identification of anatomical structures and for the surgical preparation.	2.3 ± 0.9
Q5	I followed the instructions given by the HoloPointer.	1.9 ± 0.5

## Discussion

The pre-clinical experiment was conducted to estimate the HoloPointer’s applicability and feasibility for simulated laparoscopic surgery training. The results showed advantages of using the AR technology compared to a standard condition consisting of verbal and gestural communication. Economy of movement and error rates could be improved using the HoloPointer. The visual guidance by the trainer was more effective in conveying instructions because of less ambiguity compared to verbal commands. This led to more focused instrument movements and an improved performance, e.g., more precise placement of clips, in the current pre-clinical setup.

This work’s findings are in accordance with results of related works proposing similar pointing tools. Previous studies could show improved task completion times and efficiency [10, 12], as well as improved economy of instrument movement [15] using virtual pointers. The HoloPointer differs from these approaches in terms of the displaying modality. Instead of directly superimposing the laparoscopic camera stream, the HoloPointer is displayed using AR technologies. This modality enables the addition of further functionalities, that would not be possible elsewise. For example, virtual secondary monitors can be displayed in mid-air [22]. The HoloPointer could then also be used on these virtual screens. Moreover, since AR glasses like the HoloLens, are self-contained systems, no further hardware or manipulation of existing systems is required in order to use the pointer.

The presented results are limited by the fact that the HoloPointer was only compared with a pointer-less standard method, but not with alternative pointing techniques as described in section “Related work”. Future research could examine differences between our method and previous ones. However, since most alternative pointing tools require at least one free hand of the teaching surgeon to use a mouse or a touchscreen, these methods are of disadvantage in clinical practice.

Moreover, this work did not evaluate the HoloPointer’s potential to facilitate the learning of professional vision, e.g., by investigating trainees’ gaze behavior. Such research should also be conducted in future work.

Similarly to the work of Ward et al. [13], the HoloPointer was controlled via head pointing. We argued, that this was the most applicable modality with respect to touchless or even hands-free interaction. However, it may be possible, that the pointer would be easier to control using different techniques. Minataka et al. [23] compared head pointing with gaze position tracking and foot gestures and identified head pointing as the most efficient interaction method for pointing tasks. However, hand gestures, as implemented by Feng et al. [15], were not included in their experiment. Thus, future work should examine the effects of different multi-modal interaction paradigms on the manipulation of virtual pointers.

More research should be conducted to evaluate additional features of the HoloPointer, that have not been made available during the simulator study. Options to draw lines or place stamps were not used in the current setup. Additionally, the pointer of the trainees was disabled for this evaluation. Effects of these further functionalities on the training process should be examined in the future.

## Conclusion

This article presented a virtual pointer application for laparoscopic surgery training. In contrast to related work, the proposed HoloPointer is based on AR technology using optical see-through HMDs and is manipulated hands-freely using head gestures.

The approach was evaluated regarding its applicability and feasibility for laparoscopic surgery training. A user study with ten physicians was conducted in which a trainer guided the trainees during a virtual cholecystectomy on a laparoscopic simulator. Compared to a standard condition based on verbal and gestural commands, use of the HoloPointer resulted in an improved economy of movement, less errors and an overall improved summary score. These findings indicate that the developed AR pointer is a feasible and applicable tool for laparoscopic training.

More research is required to evaluate the HoloPointer’s potential to facilitate the learning of professional vision. Future work should also focus on comparing the pointing tool to more conventional methods.

## Electronic supplementary material

Below is the link to the electronic supplementary material.

Supplementary material 1 (DOCX 15 kb)

## Data Availability

Not applicalbe.

## Code availability

Not applicalbe.
