# Supplementary material for: HoloPointer: a virtual augmented reality pointer for laparoscopic surgery training
Source: Int J Comput Assist Radiol Surg. 2020 Oct 23;16(1):161–8. doi: 10.1007/s11548-020-02272-2 (PMC7822765; doi:10.1007/s11548-020-02272-2)
Supplement: Supplementary file 1 — Supplementary material 1 (DOCX 15 kb) [file 11548_2020_2272_MOESM1_ESM.docx]

**Supplementary Table 1** Raw simulator data output. Results for each junior resident participant and mean values.

| **ID** | **Mode** | **Score** | **Total Time [s]** | **Left Instrument Path Length [m]** | **Right Instrument Path Length [m]** | **Blood Loss [ml]** | **Dissected Volume [%]** | **Ducts/Vessels Ripped [#]** | **Ducts/Vessels Burned [#]** | **Burn Damage Severity [%]** | **Left Instrument Outside View [#]** | **Left Instrument Outside View [s]** | **Right Instrument Outside View [#]** | **Right Instrument Outside View [s]** |
| --- | --- | --- | --- | --- | --- | --- | --- | --- | --- | --- | --- | --- | --- | --- |
| 1 | HoloPointer | 67,00 | 562,62 | 2,62 | 7,18 | 15,70 | 28,00 | 0,00 | 2,00 | 100,00 | 9,00 | 102,37 | 2,00 | 3,22 |
| 1 | Standard | 61,00 | 460,00 | 2,07 | 7,09 | 11,39 | 36,00 | 1,00 | 2,00 | 94,66 | 5,00 | 90,05 | 3,00 | 5,52 |
| 2 | HoloPointer | 79,00 | 520,65 | 1,91 | 6,25 | 38,33 | 24,00 | 0,00 | 1,00 | 58,61 | 8,00 | 119,05 | 1,00 | 5,98 |
| 2 | Standard | 76,00 | 615,95 | 1,96 | 7,46 | 24,62 | 34,00 | 0,00 | 0,00 | 0,00 | 11,00 | 565,55 | 3,00 | 9,65 |
| 4 | HoloPointer | 78,00 | 845,40 | 1,32 | 6,30 | 16,67 | 28,00 | 0,00 | 0,00 | 7,21 | 6,00 | 474,75 | 6,00 | 14,78 |
| 4 | Standard | 75,00 | 963,22 | 1,92 | 7,31 | 122,29 | 30,00 | 0,00 | 0,00 | 14,14 | 16,00 | 741,06 | 6,00 | 8,88 |
| 6 | HoloPointer | 87,00 | 442,50 | 1,70 | 4,44 | 11,37 | 30,00 | 0,00 | 0,00 | 1,04 | 4,00 | 352,50 | 4,00 | 4,15 |
| 6 | Standard | 73,00 | 742,75 | 4,14 | 7,53 | 96,25 | 24,00 | 0,00 | 1,00 | 40,37 | 34,00 | 450,25 | 4,00 | 2,37 |
| 7 | HoloPointer | 76,00 | 762,38 | 3,02 | 7,04 | 24,54 | 26,00 | 0,00 | 0,00 | 14,43 | 10,00 | 541,10 | 5,00 | 1,00 |
| 7 | Standard | 74,00 | 758,22 | 2,22 | 7,93 | 5,97 | 20,00 | 1,00 | 0,00 | 11,73 | 2,00 | 312,82 | 2,00 | 1,00 |
| 8 | HoloPointer | 70,00 | 609,90 | 2,80 | 6,80 | 10,88 | 32,00 | 0,00 | 2,00 | 82,54 | 8,00 | 529,52 | 2,00 | 1,53 |
| 8 | Standard | 56,00 | 740,68 | 2,75 | 8,42 | 27,60 | 30,00 | 1,00 | 2,00 | 48,17 | 5,00 | 251,42 | 6,00 | 24,53 |
| 9 | HoloPointer | 77,00 | 686,17 | 1,69 | 4,98 | 23,50 | 24,00 | 0,00 | 2,00 | 57,92 | 8,00 | 326,92 | 0,00 | 0,00 |
| 9 | Standard | 69,00 | 654,22 | 2,13 | 6,24 | 54,04 | 30,00 | 0,00 | 2,00 | 26,07 | 10,00 | 398,70 | 0,00 | 0,00 |
| **Mean** | **HoloPointer** | **76,29** | **632,80** | **2,15** | **6,14** | **20,14** | **27,43** | **0,00** | **1,00** | **45,97** | **7,57** | **349,46** | **2,86** | **4,38** |
| **Mean** | **Standard** | **69,14** | **705,00** | **2,46** | **7,43** | **48,88** | **29,14** | **0,43** | **1,00** | **33,59** | **11,86** | **401,41** | **3,43** | **7,42** |
